# Thyrotoxic Periodic Paralysis—A Misleading Challenge in the Emergency Department

**DOI:** 10.3390/diagnostics10050316

**Published:** 2020-05-18

**Authors:** Stefana Bilha, Ovidiu Mitu, Laura Teodoriu, Cristian Haba, Cristina Preda

**Affiliations:** 1 Endocrinology Department, “Grigore T. Popa” University of Medicine and Pharmacy Iasi, 700111 Iasi, Romania; stefanabilha@gmail.com (S.B.); cpreda1@yahoo.com (C.P.); 2 Cardiology Department, “Grigore T. Popa” University of Medicine and Pharmacy Iasi, 700111 Iasi, Romania; cristi.haba@gmail.com

**Keywords:** thyrotoxic periodic paralysis, hypokalemia, ventricular arrhythmia, Graves’ disease

## Abstract

Despite its’ life-threatening potential due to cardiac severe dysrhythmia in the context of severe hypokalemia, thyrotoxic periodic paralysis (TPP) often goes unrecognized. Although classically confined to young Asian men, it can occur irrespective of age, sex, and race. We report a short series of three cases of TPP as first presentation of Graves’ disease in a young Caucasian male and in two Caucasian elderly and middle-aged women, respectively. The first patient developed malignant ventricular arrhythmias due to severe hypokalemia and was defibrillated, with recovery after prompt potassium correction and administration of antithyroid agents and propranolol. The other two cases developed persistent hypokalemia despite adequate potassium chloride (KCl) repletion, with slow recovery of motor deficit and serum potassium normalization up to day 5. In the first case, long-term euthyroid state was achieved via total thyroidectomy due to the presence of a suspicious nodule that proved to be malignant. In the other two cases, medical treatment was the choice of therapy for thyrotoxicosis. None experienced recurrent TPP. Thyroid hormone evaluation is mandatory in the presence of hypokalemic paralysis, even in the absence of clinical signs of thyrotoxicosis. If TPP is confirmed, initial therapy should comprise antithyroid drugs and propranolol, besides hypokalemia correction.

## 1. Introduction

Although well described as a possible complication of Graves’ hyperthyroidism [1], thyrotoxic periodic paralysis (TPP) still represents a potential life-threatening and often misleading challenge in the Emergency Department (ED). The highest incidence (approximately 2%) is seen in the Asian population [2]. In contrast, the very low incidence of 0.1–0.2% in American Caucasians [3] makes the differential diagnosis a struggle: in Caucasians, primary forms (channelopathies) come first in the etiology of hypokalemic periodic paralysis, while other possible secondary causes besides TPP include gastrointestinal and renal pathologies, use of diuretics, etc. [2].

Although Graves’ disease is the etiology of TPP in 96% of cases, nodular goiter, subacute thyroiditis, and thyrotoxicosis factitia have also been associated with TPP [1]. Unusual is also the disproportional occurrence of TPP in men compared to women (26: 1), despite Graves’ disease being predominantly encountered in women [1]. 

Only 17–50% of TPP patients exhibit clinical signs of hyperthyroidism [1], making the diagnosis even more challenging. The sudden onset flaccid paralysis due to acute potassium shift (K^+^) in the intracellular space in the context of thyrotoxicosis can lead to cardiac arrhythmias and respiratory failure if not recognized and treated on time. The management of TPP is even more complicated by the thin line between refractory hypokalemia and rebound hyperkalemia, both predisposing to serious cardiac events [4]. Potassium chloride (KCl) supplementation is essential but often not enough to control TPP [5]. 

We report three initially unapparent cases of TPP with severe and/or refractory hypokalemia as first presentation of hyperthyroidism (Graves’ disease).

## 2. Case Presentation

**Figure 1:**
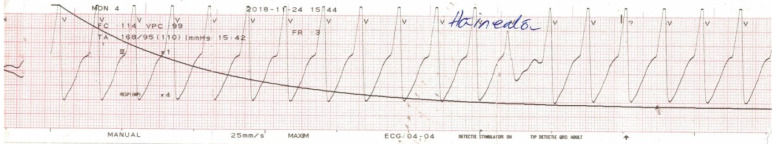
Electrocardiogram (ECG) strip showing ventricular tachycardia.

**Case one**: A 36-year-old male with negative personal medical history presented in the ED for an important malaise, a cutaneous erythematous eruption, and muscle pain which appeared after fast-food consumption. Clinical examination revealed an irregular peripheral pulse of 100 beats/min with an irregular central heart rate (HR) of 120 beats/min, blood pressure (BP) 160/80 mm Hg, systolic murmur 2/6 in the mitral area, hyperthermia (38.1 °C), and a pruritic maculopapular rash on the abdomen and lower limbs. The electrocardiogram (ECG) showed atrial fibrillation with rapid ventricular response (120/min), high R waves and negative T-waves in V4–V6, and ventricular premature beats. The echocardiography revealed a hyperkinetic heart with a slightly dilated left ventricle, moderately reduced left ventricle ejection fraction of 40%, and mild atrial dilation. Blood workup showed a low-normal serum K^+^ (3.6 mmol/L—normal range 3.5–5 mmol/L). The patient received 100 mg hydrocortisone due to the suspicion of an allergic reaction and was about to be admitted in the Cardiology Department when 1 h later he suddenly developed muscle weakness which evolved towards flaccid paralysis of the lower limbs in a matter of 30 min. Subsequently, his HR decreased to 25 bpm and the patient started experiencing syncopal episodes. Emergency ECG revealed complete atrioventricular (AV) block with escape ventricular beats that degenerated in multiple episodes of ventricular tachycardia and ventricular fibrillation (Figure 1)—in the context of severe hypokalemia of unknown etiology (serum K^+^ dropped to 1.2 mmol/L). After successful defibrillation and antiarrhythmic therapy, the patient was transferred to the Intensive Care Unit.

Emergency thoracic and abdominal computer tomography (CT) scan revealed an enlarged right thyroid lobe (51/30/65 mm), which presented a cystic mass (30.5/26/22 mm) with a calcified thin wall. Thyroid evaluation showed: (1) thyroid stimulating hormone (TSH) = 0.017 mIU/L—normal range 0.4–4 mIU/L, free thyroxine (FT4) = 2.52 ng/dL—normal range 0.89–1.76 ng/dL, free tri-iodothyronine (FT3) = 4.1 pg/mL—normal range 2.3–4.9 pg/mL), thyroid peroxidase antibodies > 1000 IU/mL—normal range <36 IU/mL) and (2) thyroid ultrasound—increased volume of the right thyroid lobe (27 mL) with a cystic nodule of 2.75 cm in its largest diameter exhibiting punctate echogenic foci and peripheral rim calcifications (TI-RADS level 5—highly suspicious). TSH-receptor antibodies (TRAb) came back positive later on during hospitalization, thus confirming the diagnosis of Graves’ disease.

**Figure 2:**
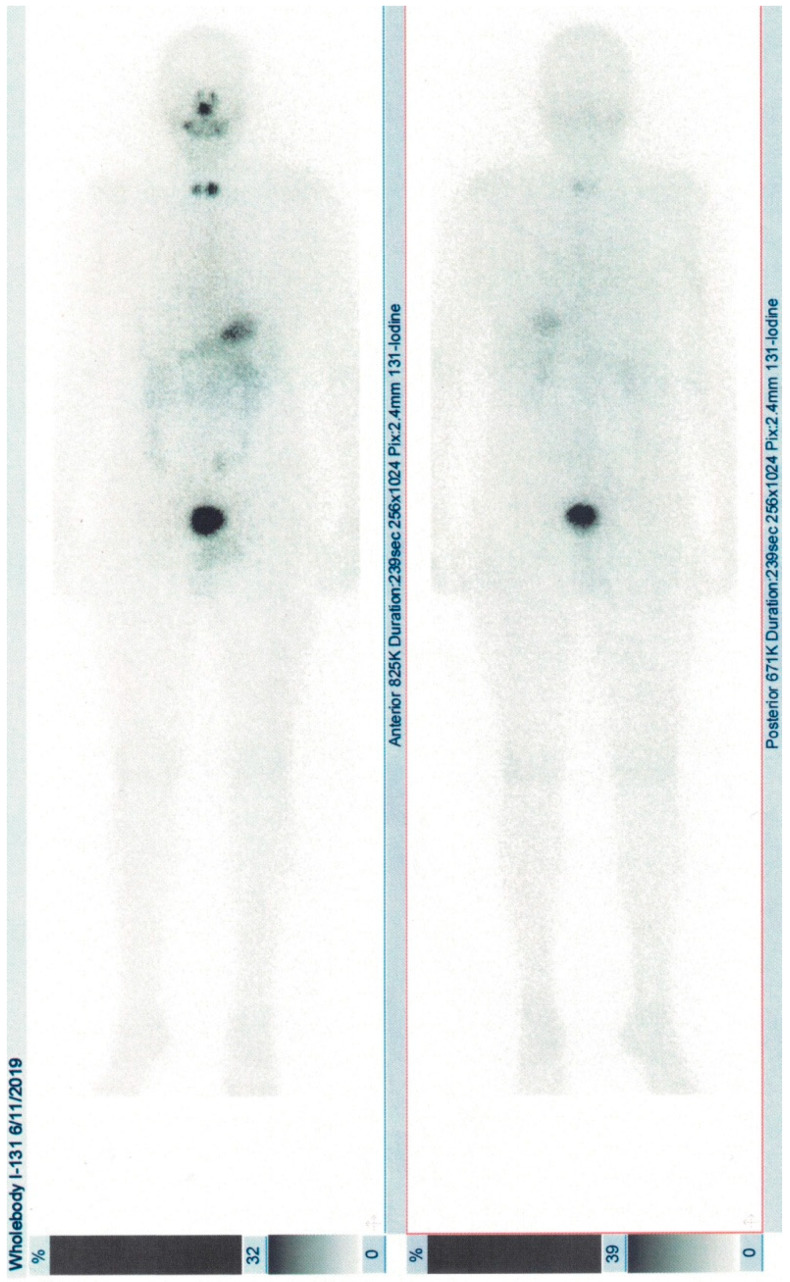
Remnant uptake in the thyroid area after iodine radiopharmaceutical washout in patient 1.

The evolution of the patient (Table 1) was favorable after prompt potassium correction and administration of antithyroid agents and nonselective beta-blockers, with rapid amelioration of cardiovascular and systemic symptoms. Due to the coexistence of Graves’ disease and a suspicious thyroid nodule, the patient subsequently underwent total thyroidectomy and the histological examination confirmed multifocal papillary carcinoma (T3bN0M0), stage I [6]. Radioiodine whole-body scintigraphy showed uptake in the cervical area and ablation with 2.56 GBq I-131 was performed (Figure 2). He is under hormonal suppressive dose of levothyroxine and ongoing observation.

**Case two**: A 69-year-old woman, with no past medical history, was brought to the ED with severe proximal muscle weakness at the level of the lower limbs accompanied by nausea and vomiting of several hours’ onset. The patient also reported weight loss, palpitations and transient episodes of muscle weakness with difficulty in standing up that began 1 month before. On presentation, she had a regular HR of 100 beats/min and 160/80 mm Hg BP. A neurological examination revealed upper limb tremor, symmetrical hyporeflexia of the knees, and paraplegia. She also had a positive Giordano sign (costovertebral angle tenderness) on the right side. Remainder of the examination was normal.

Laboratory data showed abnormal results for the white blood count (11,000/mm^3^, normal range 4000–10,000/mm^3^) and aminotransferases (ALAT = 65 U/L—normal range 2–55 U/L, ASAT = 45 U/L—normal range 5–34 U/L), and serum K^+^ (2.7 mmol/L—normal range 3.5–5 mmol/L). She had biochemical evidence of autoimmune thyrotoxicosis at the initial thyroid workup: TSH = 0.01 mIU/L—normal range 0.4–4 mIU/L, FT4 = 2.5 ng/dL—normal range 0.89—1.76 ng/dL, thyroid peroxidase antibodies = 240 IU/mL—normal range <100 U/mL, and thyroglobulin antibodies = 190 IU/mL—normal range <50 IU/mL. The thyroid ultrasound confirmed the diffuse hyperplasia of the thyroid gland (volume 43 mL). Urine culture was positive for *Escherichia coli*. The patient was admitted in the Internal Medicine Department and started on (1) thiamazole 30 mg q.i.d and propranolol 20 mg t.i.d. for the hyperthyroid state, (2) 1 g/day KCl for the hypokalemia, and (3) antibiotics for the urinary tract infection. The serum K^+^ normalized and the motor deficit regressed after 5 days of treatment (Table 1). Thyroid uptake and scintigraphy performed with 185 MBq Tc-99m pertechnetate (Tc-99m Pt) were suggestive of Graves’ disease.

She was discharged on day 25 after regaining full muscle power. Euthyroid state was achieved after 6 weeks of antithyroid medication and Graves’ disease was confirmed (positive TRAb). Our patient had no further episode of TPP.

**Case three**: 51-year-old woman was brought to the ED with suspicion of leptospirosis due to acute onset complete flaccid quadriplegia, fever, recurrent episodes of loss of consciousness, and a positive epidemiological context. Physical examination revealed normal BP 120/80 mm Hg, tachycardia 110 beats/min, complete motor deficit with abolished deep tendon reflexes but no sensory deficits at the lower extremities, and mild exophthalmos.

Initial serum potassium on presentation was 2.7 mmol/L (normal range 3.5–5 mmol/L) with normal acid–base, calcium, and magnesium status. Aminotransferases were slightly elevated (ASAT = 45 U/L—normal range 5–34 U/L and ALAT = 81 U/L—normal range 2–55 U/L). The remaining laboratory analyses, including blood count, inflammatory markers, muscle enzymes, and renal function were otherwise normal. Serological testing for leptospirosis proved negative. The patient was admitted in the Intensive Care Unit, where serum K^+^ normalized on day 5 (4 mmol/L, Table 1) and neurological signs improved after intravenous KCl supplementation.

Due to the persistence of sinus tachycardia, thyroid hormones were tested: TSH = 0.2 mIU/L—normal range 0.4–4 mIU/L, FT4 = 2.1 ng/dL—normal range 0.89–1.76, thyroid peroxidase antibodies = 500 IU/mL—normal range <100 IU/mL, and thyroglobulin antibodies = 290 IU/mL—normal range <50 UI/mL. The appearance of the thyroid parenchyma at ultrasound was heterogenous, intense hypoechoic, and with increased blood flow. The patient was transferred to the Endocrinology Department where treatment with thiamazole 30 mg q.i.d and propranolol 20 mg t.i.d. was initiated. Thyroid scintigraphy with Tc-99m Pt showed increased diffuse uptake of the thyroid gland and TRAb were found positive, thus confirming the diagnosis of Graves’ disease. The patient was discharged on day 15 after complete remission of neuromuscular symptoms and improvement of hyperthyroid signs. She did not develop any further episode of TPP. The patients have approved this research and we have obtained their consent.

## 3. Discussion

TPP is a very rare complication of thyrotoxicosis which occurs more frequently in the Asian population, typically in young Asian men [2]. The presentation circumstances in the ED are variable and polymorphic, challenging the diagnostic and therapeutic approach.

TPP is characterized by periodic, reversible attacks of muscle weakness, and paralysis due to intracellular blockade of K^+^ by the excessive thyroid hormones [7]. Thyroid hormones regulate the Na^+^-K^+^-ATP pump at a transcriptional and post-transcriptional level and also induce the release of catecholamines via beta 2 receptors, furthermore stimulating the Na^+^-K^+^-ATP pump [8]. TPP patients were shown to have 80% more Na^+^-K^+^-ATP pump activity than other thyrotoxic patients. In addition, insulin and testosterone increase the activity of the pump, which might explain the higher prevalence of TPP in males and the manifestation of symptoms after an intense workout and a high-carbohydrate meal. Mutations in the *KCNJ18* gene, which encodes for the Kir channels, have been found in approximately 33% of patients with TPP. This massive cellular influx of potassium along with decreased outflow leads to hypokalemia and TPP [9].

Thyroid hormones also play an important role in gene regulation at the level of cardiac myocytes, especially during diastole as they affect the calcium uptake into the sarcoplasmic reticulum through the calcium adenosine triphosphatase (Ca^2+^-ATPase) and phospholamban [10]. The most frequent ECG changes in patients with TPP reflect the thyrotoxicosis and hypokalemia. In addition to the typical hypokalemia pattern, studies have also described the occurrence of both conduction disorders and supraventricular and ventricular arrhythmias in patients with TPP, such as sinus arrest, second-degree AV block, P-R prolongation, right bundle branch block, and up to total AV block (as in our first case) [11].

Ventricular arrhythmias are the most dangerous cardiovascular complications of TPP and are usually seen in patients with preexisting cardiac disease. Hypokalemia can be the cause of torsade de pointes or polymorphic ventricular tachycardia, which leads to ventricular fibrillation and death if untreated [12]. Hypokalemia delays repolarization and increases diastolic depolarization of pacemaker fibers, leading to tachycardia and increasing the likelihood of ectopic beat formation. The increase in automaticity predisposes the myocardium to arrhythmias, requiring immediate defibrillation if life-threatening or unstable [13]. Our first case experienced recurrent malignant ventricular arrhythmias in the context of sudden-onset severe hypokalemia.

The usually subtle presentation of thyrotoxicosis accompanying TPP challenges the diagnosis [14]. Increased and elevated BP are more sensitive indicators of TPP than other hyperthyroid signs (fever, moist skin, exophthalmos, and goiter) [3]. In the ED, one should think of TPP if in front of the following criteria: (1) clinical and/or biochemical signs of thyrotoxicosis: a tachycardic patient with systolic hypertension and abnormal ECG (AV block) and/or low TSH together with high FT4 and FT3, (2) hypokalemia with low urinary K^+^ excretion and normal blood acid–base status (hypophosphatemia and mild hypomagnesemia can also be encountered), and (3) negative family history for periodic paralysis [1,5,15]. Laboratory tests recommended in front of acute flaccid paralysis accompanied by hypokalemia are presented in Table 2. Thyroid hormones evaluation is mandatory, even in the absence of hyperthyroid clinical signs. If thyrotoxicosis is confirmed, then measurement of thyroid antibodies including TRAb should be carried out, especially as Graves’ disease is the most common cause of TPP [1]. Although predominantly encountered in young Asian males, we together with other authors [3,16] have shown that TPP can appear irrespective of age, sex, and race, and thus, one should stay alert when facing hypokalemic paralysis.

The differential diagnosis of TPP includes primary (genetic) forms of membrane excitability disorders (channelopathies such as hypo-, normo- and hyperkalemic periodic paralysis and Andersen–Tawil syndrome, respectively) as well as secondary causes of periodic paralysis [15]. 

Primary hypokalemic periodic paralysis is caused by familial or de novo mutations in the *CACNA1S* gene (responsible for the synthesis of calcium channels) or *SCN4A* gene (encodes a critical part of sodium channels); it is characterized by hypokalemic muscle weakness episodes lasting from hours to days, usually appears under the age of 20 and is in most cases accompanied by positive family history (autosomal dominant transmission) [3,15]. Primary normo- and hyperkalemic periodic paralysis are caused by pathogenic variants in *SCN4A*, have similar clinical features as the hypokalemic form, but the age of onset is even lower, have normal or elevated serum K^+^ levels, and both exhibit myotonic discharges between attacks [15,17]. Andersen–Tawil syndrome is caused by pathogenic variants in KCNJ2 potassium channels and is characterized by episodic periodic paralysis with prolonged interictal muscle weakness and cardiac anomalies (ventricular arrhythmias and prolonged QT interval). The somatic dysmorphia (short stature, scoliosis, low-set ears, widely spaced eyes, and syndactyly) and neurocognitive deficits that are usually present (although variable) help the diagnosis, which is often made in the first two decades of life [18].

Secondary (nonfamilial) hypokalemic paralysis causes also need to be excluded: (1) hyperaldosteronism—primary or secondary; (2) urinary losses, such as diuretics, hypomagnesemia, and Bartter and Gitelman syndromes; (3) gastrointestinal losses—vomiting, diarrhea, and laxative use; and (4) other causes of intracellular K^+^ shift—recovery from diabetic ketoacidosis, beta-agonists, and metabolic alkalosis [19].

A 10-year analysis of 135 TPP patients found the presence of precipitating factors in approximately one-third of cases. The most prevalent were high carbohydrate ingestion (12%), followed by upper respiratory infections, strenuous exercise, high-salt diet, and trauma. The use of corticosteroids (1%)—such as in our first case—and beta-adrenergic bronchodilators have also been identified as trigger factors for TPP [1]. Our first patient had received hydrocortisone for the skin rash, but the other two patients did not exhibit any evident trigger factors. Corticosteroids were also reported to determine TPP in subclinical hyperthyroidism [20].

TPP usually occurs as a first manifestation of thyrotoxicosis, as only one quarter of TPP patients are already diagnosed with hyperthyroidism prior to their first attack [1]. Once euthyroid state is obtained, TPP does not usually recur (highest rate of recurrence, approximately 62.2%, is in the first 3 months) [21,22]. One-third can still experience repetitive episodes during tapering or withdrawal of antithyroid drugs [1]—probably linked to recurrence of hyperthyroidism. In the absence of antithyroid and nonselective beta-blocker medication, our patients experienced persistent hypokalemia despite adequate KCl replacement. In a prospective interventional study on 78 TPP patients, approximately one-fourth developed paradoxical hypokalemia after KCl administration. These patients had more severe symptoms of thyrotoxicosis (higher HR, BP, and serum FT4) [4]. 

The management of TPP is even more complicated as it also poses the risk of overcorrection and rebound hyperkalemia. Both paradoxical hypokalemia and rebound hyperkalemia are life-threatening situations due to potential serious cardiac arrhythmias (complete heart block and ventricular fibrillation) [23,24]. Little KCl is actually needed to correct the deficit in the absence of a true overall body depletion [24]. The risk of rebound hyperkalemia positively correlates with the dose of KCl received by the patient: administering more than 90 mEq KCl within the first 24 h or at a rate of more than 10 mEq/h poses the highest risk [5,24].

Thus, the main question in managing TPP-associated severe hypokalemia is how to find the balance between paradoxical hypokalemia and rebound hyperkalemia? Propranolol blunts the overstimulation of the Na^+^-K^+^-ATP pump, thus efficiently preventing the intracellular shift of K^+^ and reversing the paralysis without the need of excessively administering KCl [25]. Therefore, initial therapy for TPP should also include antithyroid drugs and propranolol which allow a rapid resolution of symptoms without the risk of rebound hyperkalemia [5]. Remission of thyrotoxicosis is the mainstay of therapy on the long term [26]. Other authors have also chosen surgical treatment (thyroidectomy) in the case of refractory TPP—like we did with our first patient—due the advantage of rapidly controlling the hyperthyroid state, especially if the patient has a large or suspicious goiter [21]. 

Finally, TPP is a well-known entity but since it is very rare and classically confined to a particular category of individuals, it still often goes underdiagnosed. Our experience emphasizes once more the need to bear in mind the diagnosis of TPP in every case of hypokalemic periodic paralysis, even in the absence of typical clinical signs and symptoms of thyrotoxicosis or when the clinical tableau suggests otherwise. Our first patient is even more interesting due to the debut of Graves’ disease and concomitant multifocal invasive thyroid papillary carcinoma via severe, life-threatening TPP in the absence of clinically apparent goiter or exophthalmos: to our knowledge, there are only three other papers [21,27,28] in the literature reporting the association TPP–thyrotoxicosis–thyroid carcinoma. Also, the occurrence of TPP in rather elderly Caucasian women—such as in our last two patients—makes this rare entity even rarer. We demonstrate that age, sex, and ethnicity should not be criteria to reckon on and that thyroid status evaluation should be promptly performed in order to avoid severe cardiac complications.

## 4. Conclusions

TPP is a rare, but life-threatening complication of hyperthyroidism that often goes under-recognized. In front of hypokalemic paralysis, thyroid hormone evaluation is mandatory, even in the absence of clinical signs of thyrotoxicosis and irrespective of age, sex, and race. If thyrotoxicosis is confirmed, thyroid antibodies including TRAb should be performed. Initial treatment of TPP should also include antithyroid drugs and propranolol besides KCl supplementation, as both refractory hypokalemia and rebound hyperkalemia can occur in the absence of thyroid and Na^+^-K^+^-ATP pump control. Long-term maintenance of euthyroid status is the keystone in preventing TPP recurrence.

## Figures and Tables

**Table 1:** Summary of TPP evolution in the presented patients. NI = not identified, TPP = thyrotoxic periodic paralysis.

Case	Initial Serum K^+^ (mmol/L)	Precipitating Factor	Thyrotoxic Signs	Antithyroid and Propranolol Initiation	K^+^ Normalization	TPP Severe Complications
1	1.2	Hydrocortisone	Atrial fibrillation Systolic hypertension Fever	Day 2	Day 2	Resuscitated malignant ventricular arrhythmia
2	2.7	NI	Sinus tachycardia Systolic hypertension Goiter	Day 1	Day 5	None
3	2.7	NI	Tachycardia Exophthalmos	Day 5	Day 5	None

**Table 2:** Laboratory workup for hypokalemic acute flaccid paralysis [5,15].

Laboratory Tests Suggestive for TPP
Serum electrolytes: hypokalemia < 3.5 mmol/L Arterial blood gas analysis—normal acid–base balance Serum PO4, Mg: hypophosphatemia, hypomagnesemia Urine potassium excretion: urinary K^+^ <25 mmol/L or urinary K^+^-to-creatinine ratio <13 Thyroid function tests: low TSH, increased FT4, increased FT3

PO4 = phosphate, Mg = magnesium, K^+^ = potassium, TSH = thyroid stimulating hormone, FT4 = free thyroxine, FT3 = free tri-iodothyronine, TPP = thyrotoxic periodic paralysis.
